# Normative data and correlation between dynamic knee valgus and neuromuscular response among healthy active males: a cross-sectional study

**DOI:** 10.1038/s41598-020-74177-8

**Published:** 2020-12-02

**Authors:** Luis Llurda-Almuzara, Albert Pérez-Bellmunt, Carlos López-de-Celis, Ramón Aiguadé, Roberto Seijas, Oriol Casasayas-Cos, Noe Labata-Lezaun, Pedro Alvarez

**Affiliations:** 1 C/Josep Trueta s/n, Sant Cugat del Vallès, Facultat de Medicina i Ciències de la Salut, Universitat Internacional de Catalunya, 08195 Barcelona, Spain; 2 Department of Orthopaedic Surgery, Hospital Quirónsalud, Barcelona, Spain; 3 Universitat de Lleida, Lleida, Spain

**Keywords:** Anatomy, Risk factors

## Abstract

The dynamic knee valgus (DKV) during different sport maneuvers has been widely described as risk factor to develop an anterior cruciate ligament injury. Hip and knee muscles seem to have a crucial role to prevent the dynamic knee valgus. This study aimed to give normative and correlational data about DKV and hip and knee neuromuscular response (NMR) among healthy active males. The hypothesis is that DKV could be correlated with hip NMR. A cross-sectional correlational study. Research Anatomy Laboratory. The study was carried out among 50 active, non-injured males. Dynamic Knee-Valgus angle and lower limb posterior chain muscles Neuromuscular Response. DKV was measured using Kinovea software during a Single-Legged Drop Jump test and NMR was measured using tensiomyography and myotonometry for gluteus maximum, biceps femoris, semitendinosus, lateral and medial gastrocnemius. Right and left limbs were both performed and analyzed independently. No significant correlation was observed between DKV and hip and knee muscles NMR. This study shows normative and correlational data about dynamic knee valgus, tensiomyography and myotonometry for healthy and active males. The DKV control seems to be non-correlated with isolated hip and knee muscles NMR so this suggests it is more about Central Nervous System activity than about isolated muscles NMR.

## Introduction

The anterior cruciate ligament (ACL) injury is a common injury in general population with an annual incidence of 68.6 per 100,000 person-years^1^. This data is significantly higher among professional athletes (ranging from 150 to 370 injuries per 100,000 person-years) and amateur athletes (ranging from 30 to 162 injuries per 100,000 person-years)^2^. Several authors have suggested that most of these injuries occur by non-contact mechanisms^3^. Video-analysis studies have described the dynamic knee valgus (DKV) during landing, pivoting or deceleration maneuvers as the most common ACL injury in sports as football, basketball or handball^4–7^. The DKV is a medial collapse of the knee resulting in a hip adduction and internal rotation, tibial abduction and medial knee displacement which increases the ACL strain^8^. Many studies have measured the dynamic knee valgus in the sagittal plane using 2D video-analyisis^9–12^. The main cause of the DKV are neuromuscular control deficits and, therefore, injury prevention and rehabilitation strategies are currently focusing on improving neuromuscular control in order to avoid this injury mechanisms^13^.

Within neuromuscular system, the hip and the knee muscles seem to have a crucial role to prevent the dynamic knee valgus. Specifically, athletes with poor glutes and hamstring activation are more likely to collapse the knee during a landing and then, potentially increasing the risk of non-contact ACL injury^14^. In fact, a protocol for neuromuscular activation of the abductors and external hip rotators during warm-up has recently been shown to reduce the DKV by 53–63% in youth male soccer players^15^. The relation between hip strength and the dynamic knee valgus seems to be clear^16^, but a possible relation between DKV and neuromuscular response (NMR) as contraction time, stiffness, tone or muscular displacement remain unstudied. The NMR is a combination of biomechanical parameters of the muscle tissue that measured by two different methodologies, the MyotonPro device (mytonPro, Myoton Ltds., Estonia) and Tensiomyography (TMG-BMC. Ljubljana, Slovenia). While the Myoton provides information about the tone, stiffness, relaxation and elasticity of the muscle^17,18^, the TMG provides information about muscle stiffness or tone, contraction velocity, type of predominant skeletal muscle fibers, or muscle fatigue^19–22^.

The aim of this study was: (1) to give normative data about DKV and NMR among active, non-injured males and (2) to study a possible relation between the dynamic knee valgus and hip and knee neuromuscular response as contraction time, stiffness, tone or muscular displacement. We hypothesized that DKV angle could be correlated with hip and knee NMR parameters.

## Materials and methods

### Ethical considerations

Approval was obtained from the local ethics committee (CER-UIC-Barcelona; study code: CBAS-2018-17) and the study was conducted in accordance with the declaration of Helsinki. Informed consent was obtained from all individual participants included in the study.

### Participants

The sample size was calculated using the *Calculadora de Grandària Mostral* (GRANMO v7.12) software based on McCurdy et al.^23^ results to detect correlation coefficients of 0.41 or greater at α = 0.05 and β = 0.20. A sample size of minimum 50 participants was needed. The participants recruited for the present study were 50 healthy young adults (between 18 to 29 years old). A convenience sample was recruited by informative posters put up across University asking for participants.

The inclusion criteria were: (1) to have signed the informed consent and (2) to do physical activity at least three times per week.

Participants were not eligible to participate if they: (1) had sustained a lower limb injury for the last year or (2) did not understand the information given or (3) had a history of ACL injury.

Fifty healthy males participated in this study. Variables with normal distribution are described as mean ± standard deviation and variables with abnormal distribution are described as median ± interquartile range. Baseline characteristics and DKV values of the sample are shown in Table 1.

**Table 1:** Baseline characteristics.

Variable	N	Mean ± SD [95% CI]
Age (years)	50	21.84 ± 5.25 [20.38, 23.29]
Weight (kg)	50	68.96 ± 11.84 [65.67, 72.24]
Height (cm)	50	182.37 ± 19.68 [176.91, 187.82]
BMI	50	21.69 ± 3.70 [20.66, 22.71]
Physical activity (days/week)	50	3.04 ± 1.52 [2.61, 3.46]
Right dynamic valgus (˚)	50	12.06 ± 7.60 [9.95, 14.16]
Left dynamic valgus (˚)	50	9.50 ± 13.80^a^ [5.67, 13.32]

### Experimental design

A cross-sectional, correlation study examining healthy active males were used for this study. The variables were dynamic knee valgus and hip and knee neuromuscular response.

### Dynamic knee valgus

A two-dimensional video-analysis was used to measure the knee-valgus/varus frontal-plane projection angle (FPPA) during a Single-Legged Drop Jump test^12,24^. The knee valgus/varus frontal-plane angle was defined as the angle between (1) the ankle midpoint, (2) the patella midpoint and (3) the projection line between the patella midpoint and the anterior superior iliac spine^8^. The Single-Legged Drop Jump test is a test where the subjects were asked to do a single-legged drop from a 50 cm box, to jump immediately as high as possible and to land with only one leg. Subjects had to do it with the right leg firstly and with the left leg secondly.

**Figure 1:**
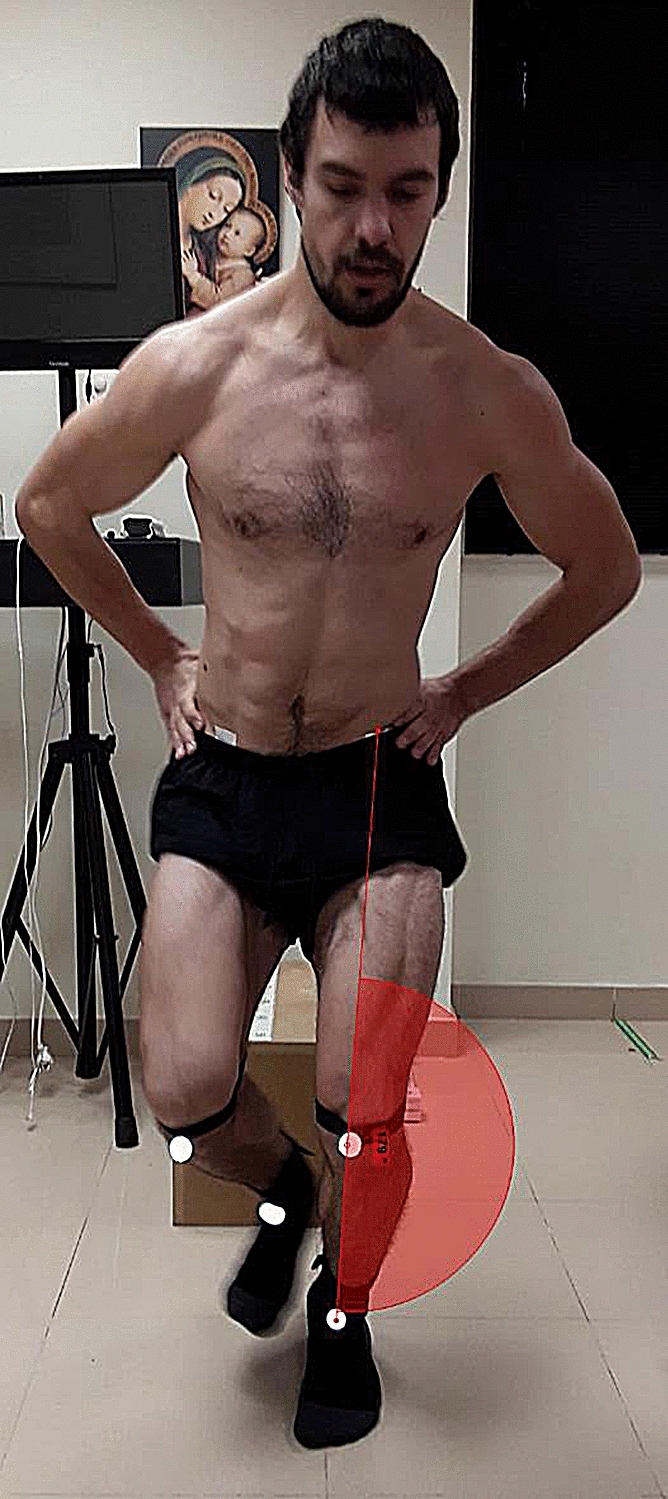
Dynamic knee valgus with Kinovea software.

The *Kinovea software* v0.8.26 (Kinovea open source project under GPLv2) was used to quantify the FPPA^25^. The video camera was placed at the height of 50 cm from the bottom, three meters anterior to the subject and the maximum FPPA during the Single-Legged Drop Jump test was recorded (Fig. 1).

### Neuromuscular response (NMR)

The methodologies for tensiomyography (TMG) and Myoton assessment were identical in both sides, and values were obtained by the same investigator, who had experience with both tools. All subjects were instructed to come for measurements in the following conditions as previous research from Alvarez-Diaz et al^26^ suggested: (1) resting, with no previous high intensity exercise and no intake of energy drinks within lasts 48 h; (2) no alcohol or caffeine at least 3 h before measurements; and (3) no food intake at least 2 h before measurements. The environmental conditions of the room are controlled (temperature: 21–23 ºC and humidity: 40–50%) so that they were the same throughout the process.

**Figure 2:**
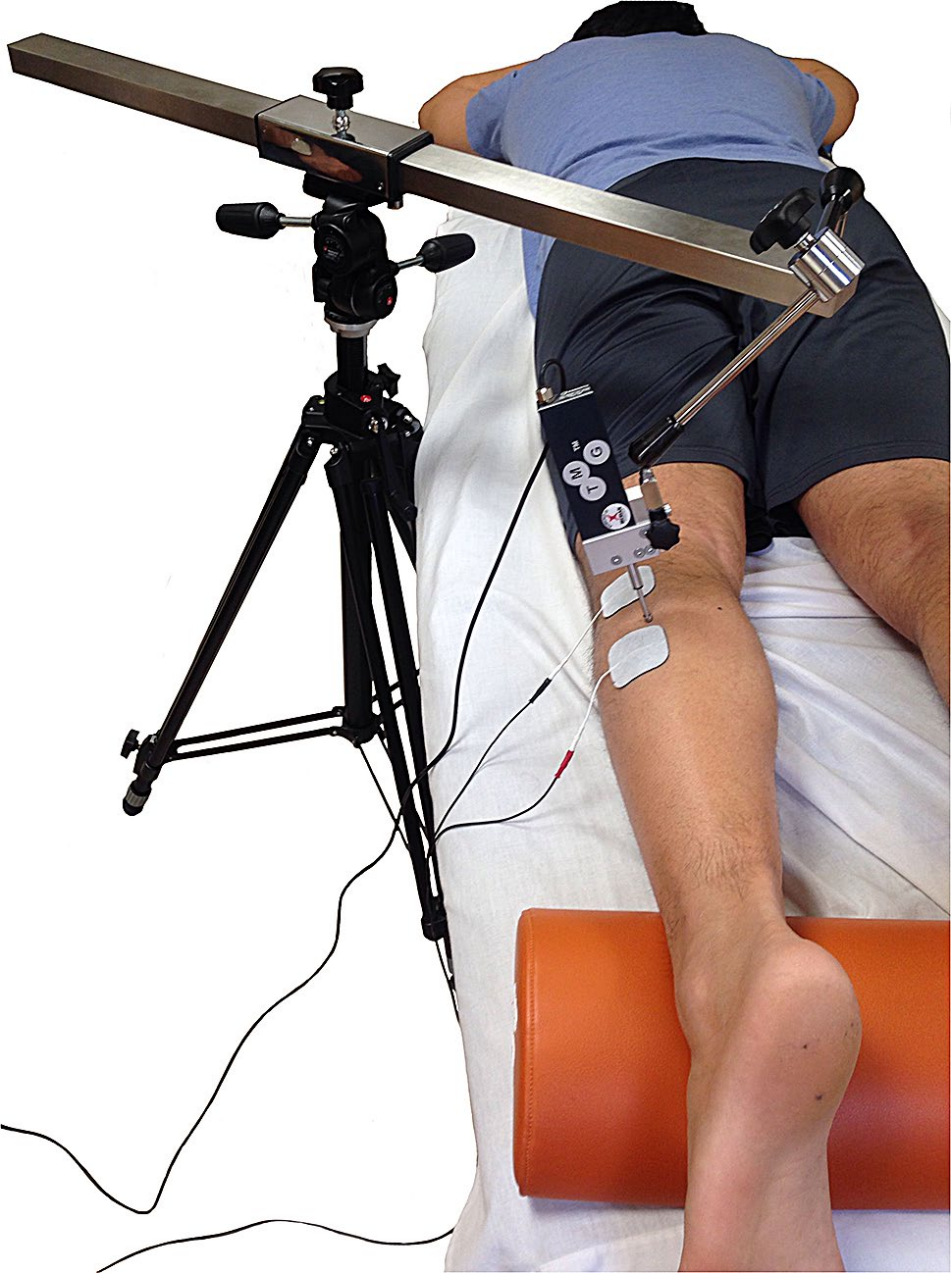
Tensiomyography.

The TMG (Fig. 2) has shown a good inter-observer, intra-session and between-day reliability for lower limb muscles^21,26–30^. The TMG provides data about the muscle belly radial displacement, which is called maximal displacement (Dm). It is expressed in mm and it informs about muscle stiffness^26,31^. Moreover, TMG provides data about the delay time (Td), which is the time between the initiation and 10% of *D*m; the contraction time (*T*c), which is the time between 10 and 90% of *D*m; the sustained time (*T*s) is the time in which the muscle response remains > 50% of *D*m; and the half-relaxation time (*T*r), which is the time in which the muscle response decreases from 90 to 50% of *D*m^26^. From all this parameters, *Dm* and *Tc* are the two most utilized among research^29^.

The measurement methods, protocol and anatomical localization of the sensors were standardized for all subjects and established according a previous study from Rey et al^21^ and Álvarez et al^26^. All measurements were obtained at rest in prone position. The self-adhesive electrodes (TMG electrodes, TMG-BMC d.o.o. Ljubljana, Slovenia) were placed equidistant to the measuring point^26^. The digital transducer Dc–Dc Trans-Tek (GK 40, Panoptik d.o.o., Ljubliana, Slovenia) was placed perpendicular to the muscle belly between both electrodes^26^. A TMG-100 System electrostimulator (TMG-BMC d.o.o., Ljubljana, Slovenia) with a pulse of 1 ms and initial amplitude of 50 mA was used for the electrical stimulation^26^. For each test, amplitude was progressively increased by 10 mA increments until there was no further increase in *D*m or maximal stimulator output (110 mA)^26^.

The tensiomyography test was carried out in both glutes maximum (Gmax), biceps femoris (BF), semitendinosus (ST), gastrocnemius medialis (GM) and gastrocnemius lateralis (GL).

**Figure 3:**
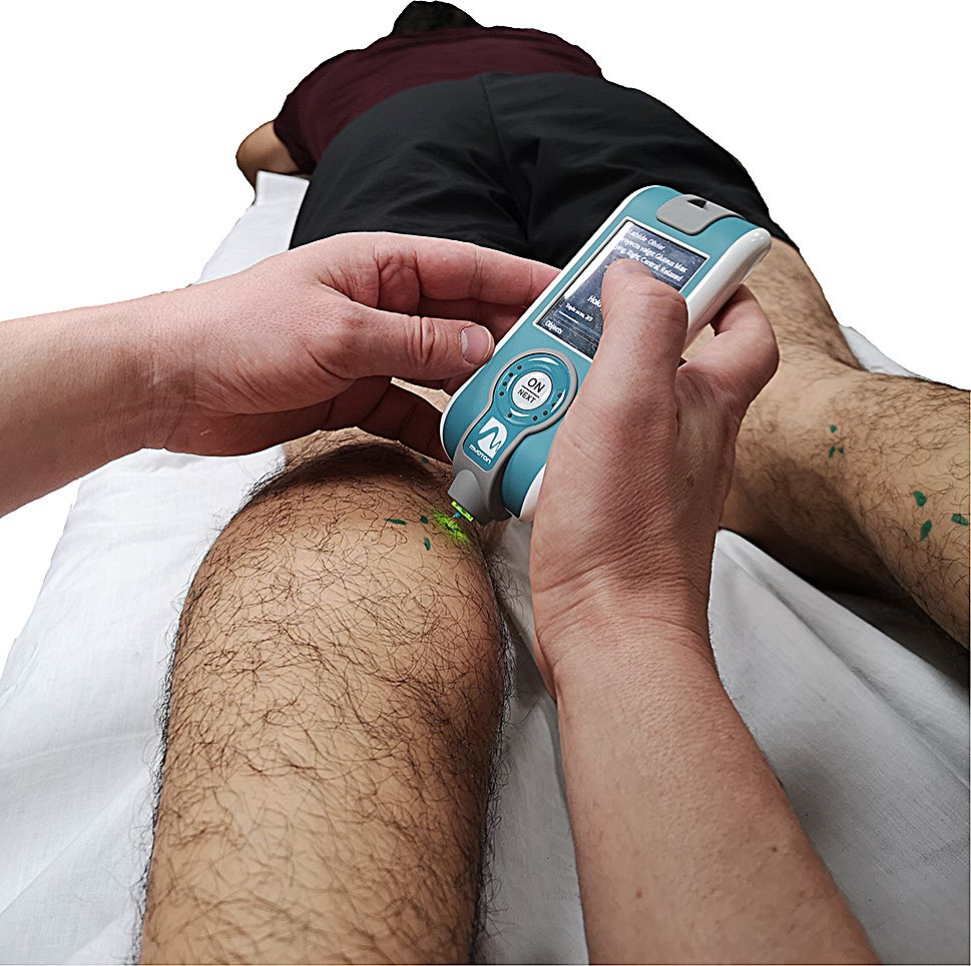
MyotonPro.

The MyotonPro (mytonPro, Myoton Ltds., Estonia) (Fig. 3) uses a portable device to measure the deformation properties of natural damped oscillations produced due to a short (15 ms) mechanical tap to the surface of the skin with a good reliability for lower limb muscles^32–35^. The parameters obtained by MyotonPro were (1) Frequency (natural oscillation frequency characterizing the *tone* of the muscle in resting state), (2) Displacement (logarithmic decrement of natural oscillation, characterizing *elasticity*), (3) Dynamic *stiffness*, characterizing the muscle resistance to contraction, (4) ratio of relaxation time to deformation time, characterizing *creep* and (5) mechanical stress *relaxation* time. The measurement methods, protocol and anatomical localization of the sensors were standardized for all subjects and established according to previous studies^35^. All measurements were obtained at rest in the prone position with the sensor device placed in the middle of the muscle belly.

(identified by manual palpation) and angled 90° to the skin surface. For each test, the MyotonPro device took three taps and the mean of each parameter was obtained. Measurement was valid only if the variation between three taps was less than 5%.

The MyotonPro test was carried out in both glutes maximum (Gmax), biceps femoris (BF), semitendinosus (ST), gastrocnemius medialis (GM) and gastrocnemius lateralis (GL).

### Statistical analysis

SPSS v.20 (IBM Corp., Armonk, N.Y., USA) was used for the statistical analysis. Quantitative variables and differences are expressed mean and standard deviation (SD) or median ± interquartile range (IQR). The level of significance was set at α = 0.05 and confidence interval limits at 95%. The suitability of using parametric or non-parametric tests was verified with the Shapiro–Wilk test. The correlation analysis was performed using the Pearson correlation coefficient for parametric variables or Spearman's rank correlation coefficient for non-parametric variables.

### Ethical approval

The institutional ethics committee approved the present study (CBAS-2018-17). Funding: There is no funding source.

## Results

Descriptive data for variables measured in this study are shown at Tables 2 and 3 for neuromuscular response measured by Tensiomyography and MyotonPro respectively. Only contraction time and maximal displacement (for TMG) and tone and stiffness (for Myoton) are shown on the tables because they are the most clinically relevance measures.

**Table 2:** Descriptive data from tensiomyography.

Muscle	Variable	N	Mean ± SD [95% CI]
Left gluteus maximus	Tc	50	33.97 ± 8.09 [31.73, 36.21]
	Dm	50	7.32 ± 2.60 [6.59, 8.04]
Left biceps femoris	Tc	50	41.49 ± 12.62 [37.99, 44.98]
	Dm	50	6.99 ± 2.25 [6.36, 7.61]
Left semitendinosus	Tc	50	39.20 ± 7.82 [37.03, 41.36]
	Dm	50	9.35 ± 2.65 [8.61, 10.08]
Left lateral gastrocnemius	Tc	50	28.75 ± 36.12^a^ [18.73, 38.76]
	Dm	50	5.10 ± 2.39 [4.43, 5.76]
Left medial gastrocnemius	Tc	50	25.61 ± 6.35^a^ [23.84, 27.37]
	Dm	50	3.39 ± 1.51[2.97, 3.80]
Right gluteus maximus	Tc	50	32.85 ± 8.39 [30.52, 35.17]
	Dm	50	7.48 ± 4.72 [3.98, 10.97]
Right biceps femoris	Tc	50	42.73 ± 13.37 [39.02, 46.43]
	Dm	50	7.09 ± 4.32^a^ [5.89, 8.28]
Right semitendinosus	Tc	50	39.75 ± 6.71^a^ [37.89, 41.60]
	Dm	50	8.92 ± 2.85 [8.13, 9.70]
Right lateral gastrocnemius	Tc	50	27.16 ± 31.61^a^ [18.39, 35.92]
	Dm	50	5.26 ± 2.26 [4.63, 5.88]
Right medial gastrocnemius	Tc	50	27.72 ± 8.08^a^ [25.48, 29.95]
	Dm	50	3.90 ± 1.75 [3.41, 4.38]

*SD* standard deviation*,* *Tc* contraction time*, Dm* maximal displacement.

^a^Median ± interquartile range*.*

**Table 3:** Descriptive data from myotonometry.

Muscle	Variable	N	Mean ± SD [95% CI]
Left gluteus maximus	Tone	50	15.13 ± 1.74 [14.64, 15.61]
	Stiffness	50	157.12 ± 22.42 [150.90, 163.33]
Left biceps femoris	Tone	50	15.13 ± 1.74 [14.64, 15.61]
	Stiffness	50	271.51 ± 45.84 [258.80, 284.21]
Left semitendinosus	Tone	50	15.95 ± 1.64 [15.49, 16.40]
	Stiffness	50	282.22 ± 39.78 [271.19, 293.24]
Left lateral gastrocnemius	Tone	50	15.73 ± 1.97 [15.18, 16.27]
	Stiffness	50	265.00 ± 61.50^a^ [247.95, 282.04]
Left medial gastrocnemius	Tone	50	15.45 ± 1.85 [14.93, 15.96]
	Stiffness	50	261.45 ± 33.90 [252.05, 270.84]
Right gluteus maximus	Tone	50	10.63 ± 0.65 [10.44, 10.81]
	Stiffness	50	164.00 ± 27.00^a^ [156.51, 171.48]
Right biceps femoris	Tone	50	15.30 ± 2.02 [14.74, 15.85]
	Stiffness	50	272.22 ± 51.25 [258.01, 286.42]
Right semitendinosus	Tone	50	16.17 ± 1.90 [15.64, 16.69]
	Stiffness	50	286.96 ± 48.01 [273.65, 300.26]
Right lateral gastrocnemius	Tone	50	15.61 ± 1.93 [15.07, 16.14]
	Stiffness	50	272.49 ± 42.39 [260.74, 284.23]
Right medial gastrocnemius	Tone	50	15.65 ± 1.82 [15.14, 16.15]
	Stiffness	50	263.14 ± 34.36 [253.61, 272.66]

*SD* standard deviation*,* *Tc* contraction time*, Dm* maximal displacement.

^a^Median ± interquartile range*.*

The correlation analysis showed no significant correlation between DKV and any NMR parameter (p > 0.05; r < 0.3). Neither the Myoton nor the Tensiomyography parameters measured in both glutes maximus, biceps femoris, semitendinosus, lateral gastrocnemius and medial gastrocnemius were significantly correlated with frontal plane knee angle during the Single-Legged Drop Jump test (“Annex 1”). Moreover, no correlation was found between DKV or NMR with baseline characteristics as weight, height, BMI, days of physical activity and age. Complete correlation analysis with all TMG and MTT parameters is available on Supplemental File 1.

## Discussion

### Normative data about FPPA

The principal aim of this study was to show normative data for FPPA and NMR among healthy, non-injured population. As shown, males on this study had a FPPA mean ± SD of 12.06 ± 7.60 for right leg and a median ± IQR of 9.5 ± 13.8 for left leg.

Herrington et al.^36^ measured the FPPA among very similar population from this study. However, sample from this study seems to have greater valgus angle than those from Herrington. They found a mean FPPA of 4.9˚ for both legs in a male population.

Comparing data from this study to other types of population, Munro et al.^37,38^ showed a FPPA much smaller among female basketball and football players in two different studies. It suggests that athletes and/or females had smaller FPPA than active and healthy males. It could be easily explained because of the regular strength and motor control training that athletes do. It surely helps to avoid greater FPPA.

Greater FPPA and then, DKV during jumping have been widely described as biomechanical alteration that could lead to an ACL injury^39^, the responsibility to avoid this condition remains confuse. It is known that hip and knee muscle function have a crucial role controlling the knee position^40,41^. However, the current evidence suggests that it is not only a question about muscle characteristics but about motor control^13,15^. Motor control means the ability to control movements during functional activities such as jumping, running, squatting, pivoting, etc^42^. This obviously includes the Central Nervous System (CNS) into the game.

### Normative data about NMR

The TMG have been recently used to evaluate fatigue-induced muscle changes^43,44^, to assess muscle impairments^45^, to evaluate and quantify painful muscle trigger points^46^ and to corroborate rehabilitation muscle adaptations along the time^47^ in specific populations. These studies showed the TMG as a really clinical useful tool to evaluate muscle characteristics and changes but, due to the specificity of the population, it is not possible to compare them with data from this study^48^. No studies showing normative data for healthy and active young adults were found. This study shows normative data for maximal displacement (*D*m) and the contraction time (*T*c) for all principal lower limb muscles among healthy and active population.

Many studies have studied neuromuscular response on biceps femoris in healthy population. Sánchez-Sánchez et al.^49^ found a mean of 5.7 mm Dm and 28.45 ms Tc in elite futsal players. Other study from Sánchez-Sánchezet al.^50^ found a mean of 6.87 mm Dm and 37.36 ms Tc in u18 soccer players. García-García^51^ found a mean of 6.8 mm Dm and 34.2 ms Tc in elite cyclists. Álvarez-Díazet al.^26^ found a mean of 4.6 mm Dm and 24.5 ms Tc in soccer players. Zubacet al.^52^ found a mean of 6.2 mm Dm and 42.1 ms Tc in a healthy and active population very similar to those from the current study which found a mean of 7.1 mm Dm and 42 ms Tc. It suggests that biceps femoris Dm and Tc differs between different types of population showing lower values for athletes than for active people. Only one study^53^ was found showing semitendinosus TMG data for healthy people and it showed 9.6 mm Dm and 35.4 ms Tc in contrast with 9.1 mm Dm and 39 ms Tc showed in the present study.

Same results have been found for gastrocnemius medialis and gastrocnemius lateralis. Zubacet al.^52^ found a mean of 4.5 and 4 mm Dm and 29.2 and 30 ms Tc for gastrocnemius lateralis and medialis respectively among a very similar population from this study which found 5.2 and 3.6 mm Dm and 27 and 26 ms Tc for lateralis and medialis gastrocnemius respectively. However, Alvarez-Diaz et al.^53^ found lower values of Dm and Tc for soccer players suggesting that gastrocnemius neuromuscular response differ between athletes and active people showing lower values for athletes than for active people.

No studies were found providing TMG data of the gluteus maximum in non-injured people so data from this study cannot be compared. This is the first study showing TMG data from a crucial muscle to control and protect the knee as it is the gluteus maximus.

On the other hand, the myotonometry usefulness in order to evaluate the muscle stiffness seems to be doubtless^32^. Moreover, some studies have linked stiffness with sport-related injuries^54–56^. Only one study from Ditroilo et al.^57^ was found in order to compare myotonometry data with population from the current study. It only measured biceps femoris neuromuscular response and it obtained a mean of 15.8 Hz for tone and 328.3 N m^-1^ for stiffness. This data is very similar to these from this study that found 15.3 Hz for tone and 271 N m^-1^ for stiffness. This is the first study showing normative data for stiffness and tone for all principal lower limb muscles among healthy and active population.

### Correlation analysis

TMG and Myoton are both really interesting tools that have demonstrated its reliability to measure the neuromuscular response in an isolated muscle^27,32^. However, they do not take into account the Central Nervous System and his importance to produce a muscle strong and quick contraction.

So, regarding on the second aim of this study, the ability to control the knee valgus during a Single-Legged Drop Jump was not correlated (p > 0.05; r < 0.3) with isolate neuromuscular response. This supports the hypothesis that dynamic knee valgus control is more about CNS function than isolated NMR. Exercises aiming improve isolated muscle such as glute maximum or hamstrings may have a role on first states of the rehabilitation. But, if the aim is to control knee valgus, in order to prevent ACL injuries CNS training may be the key.

### Limitations

The biggest limitation of this study is the little specificity of the sample. Future research should be carried out with people or athletes at ACL injury risk to compare the data and to evaluate a possible correlation between DKV and NMR. The present study did not found a relation but maybe, this relation could be appear in a sample more likely to suffer an ACL injury due to sport type or injury history, for example.

### Strengths

This is the first study to show normative TMG data for gluteus maximus. No study has provided normative data about MTT parameters in healthy and active sample so it is the first study providing it. In fact, two different and novel techniques have been used in order to inform about the neuromuscular response of posterior chain muscles. Furthermore, this is the first study aiming to explore the relation between dynamic knee valgus and neuromuscular response of these muscles.

## Conclusions

This study shows normative data about dynamic knee valgus, tensiomyography and myotonometry for healthy and active population.

Moreover, this study found no correlation between DKV and NMR and this could be explained because of the influence of Central Nervous System. To control dynamic knee valgus during sport maneuvers such as single leg jumps is crucial in order to prevent sport-related injuries as anterior cruciate ligament. This study suggests that Central Nervous System activity is more important than isolated hip and knee muscles response in order to control this condition. So, anterior cruciate ligament injury prevention exercises should focus on motor control and CNS activity more than improving muscle strength and/or muscle tone.

## Annex 1

**Table:** 

Correlations table									
Muscle	Right/left			Device	Variable	Statistic		Dynamic left knee valgus	Dynamic right knee valgus
Gluteus max	Right			Tensiomyography	Tc	Correlation coefficient			0.219^a^
						Sig			0.127^a^
					Dm	Correlation coefficient			0.161^a^
						Sig			0.263^a^
				Myotonometry	Tone	Correlation coefficient			0.181^a^
						Sig			0.212^a^
					Stiffness	Correlation coefficient			0.206^b^
						Sig			0.156^b^
	Left			Tensiomyography	Tc	Correlation coefficient		0.121^a^	
						Sig		0.414^a^	
					Dm	Correlation coefficient		− 0.062^a^	
						Sig		0.674^a^	
				Myotonometry	Tone	Correlation coefficient		0.031^a^	
						Sig		0.836^a^	
					Stiffness	Correlation coefficient		− 0.118^a^	
						Sig		0.431^a^	
Bíceps femoris	Right			Tensiomyography	Tc	Correlation coefficient			0.218^a^
						Sig			0.128^a^
					Dm	Correlation coefficient			− 0.016^b^
						Sig			0.912^b^
				Myotonometry	Tone	Correlation coefficient			− 0.081^a^
						Sig			0.581^a^
					Stiffness	Correlation coefficient			− 0.102^a^
						Sig			0.487^a^
	Left			Tensiomyography	Tc	Correlation coefficient		− 0.058^a^	
						Sig		0.697^a^	
					Dm	Correlation coefficient		− 0.233^a^	
						Sig		0.112^a^	
				Myotonometry	Tone	Correlation coefficient		− 0.201^a^	
						Sig		0.176^a^	
					Stiffness	Correlation coefficient		− 0.173^a^	
						Sig		0.244^a^	

*Tc *contraction time, *Dm *muscle displacement, *Sig. *significance at α=95%.

^a^Pearson.

^b^Rho Spearman.

## Supplementary information

Supplementary Information.
