# Supplementary material for: Normative data and correlation between dynamic knee valgus and neuromuscular response among healthy active males: a cross-sectional study
Source: Sci Rep. 2020 Dec 2;10:17206. doi: 10.1038/s41598-020-74177-8 (PMC7710730; doi:10.1038/s41598-020-74177-8)
Supplement: Supplementary file 1 — Supplementary Information. [file 41598_2020_74177_MOESM1_ESM.docx]

**Supplementary File 1**

**Title of the manuscript:** Normative data and correlation between dynamic knee valgus and neuromuscular response among healthy active males: a cross-sectional study

**Authors**: Luis LLURDA-ALMUZARA^1,*,+^, Albert PÉREZ-BELLMUNT^1,+,*^, Carlos LÓPEZ-DE-CELIS^1^*, Ramón AIGUADÉ^3^, Roberto SEIJAS^1,2^, Oriol CASASAYAS-COS^1^, Noe LABATA-LEZAUN^1^, Pedro ALVAREZ^1,2^

**Affiliations:**

1. Universitat Internacional de Catalunya, Barcelona, Spain.
2. Department of Orthopaedic Surgery, Hospital Quirónsalud, Barcelona, Spain.
3. Universitat de Lleida, Lleida, Spain.

***Correspondings Authors**

- Dr Albert Pérez Bellmunt. Facultat de Medicina i Ciències de la Salut. Universitat Internacional de Catalunya. C/ Josep Trueta s/n. 08195 Sant Cugat del Vallès, Barcelona, Spain. Tel: +34 935 04 20 14. E-mail: [aperez@uic.es](mailto:aperez@uic.es)

- Sr. Luis Llurda-Almuzara. Facultat de Medicina i Ciències de la Salut. Universitat Internacional de Catalunya. C/ Josep Trueta s/n. 08195 Sant Cugat del Vallès, Barcelona, Spain. Tel: +34 935 04 20 14. Email: [lllurda@uic.es](mailto:lllurda@uic.es)

- Dr. Carlos López-de-Celis. Facultat de Medicina i Ciències de la Salut. Universitat Internacional de Catalunya. C/ Josep Trueta s/n. 08195 Sant Cugat del Vallès, Barcelona, Spain. Tel: +34 935 04 20 14. E-mail: [carlesldc@uic.es](mailto:carlesldc@uic.es)

| **Parametric correlations between right DKV and right TMG parameters** | | |
| --- | --- | --- |
|  | Pearson Correlation | Sig. |
| Gmax_right_Tc | 0,219 | 0,127 |
| Gmax_right_Ts | -0,008 | 0,954 |
| Gmax_right_Tr | 0,028 | 0,846 |
| Gmax_right_Dm | 0,161 | 0,263 |
| Gmax_right_Td | -0,013 | 0,927 |
| BF_right_Tc | 0,218 | 0,128 |
| BF_right_Ts | 0,119 | 0,410 |
| BF_right_Tr | -0,029 | 0,841 |
| BF_right_Dm | -0,155 | 0,281 |
| BF_right_Td | -0,133 | 0,356 |
| ST_right_Tc | -0,180 | 0,210 |
| ST_right_Ts | 0,139 | 0,334 |
| ST_right_Tr | -0,037 | 0,799 |
| ST_right_Dm | -0,051 | 0,728 |
| ST_right_Td | -0,089 | 0,540 |
| GasLat_right_Tc | -0,187 | 0,194 |
| GasLat_right_Ts | 0,260 | 0,068 |
| GasLat_right_Tr | 0,138 | 0,341 |
| GasLat_right_Dm | -0,180 | 0,212 |
| GasLat_right_Td | -0,095 | 0,511 |
| GasMed_right_Tc | -0,017 | 0,909 |
| GasMed_right_Ts | 0,148 | 0,305 |
| GasMed_right_Tr | -0,001 | 0,993 |
| GasMed_right_Dm | 0,015 | 0,918 |
| GasMed_right_Td | -0,147 | 0,312 |

| **Non-parametric correlations between right DKV and right TMG parameters** | | |
| --- | --- | --- |
|  | Spearman Correlation | Sig. |
| Gmax_right_Tc | 0,214 | 0,135 |
| Gmax_right_Ts | 0,074 | 0,608 |
| Gmax_right_Tr | 0,114 | 0,431 |
| Gmax_right_Dm | 0,152 | 0,291 |
| Gmax_right_Td | 0,030 | 0,834 |
| BF_right_Tc | 0,211 | 0,142 |
| BF_right_Ts | 0,041 | 0,780 |
| BF_right_Tr | -0,041 | 0,778 |
| BF_right_Dm | -0,016 | 0,912 |
| BF_right_Td | -0,034 | 0,814 |
| ST_right_Tc | -0,178 | 0,217 |
| ST_right_Ts | 0,075 | 0,607 |
| ST_right_Tr | -0,086 | 0,551 |
| ST_right_Dm | -0,099 | 0,494 |
| ST_right_Td | -0,030 | 0,835 |
| GasLat_right_Tc | -0,203 | 0,157 |
| GasLat_right_Ts | 0,181 | 0,207 |
| GasLat_right_Tr | 0,148 | 0,306 |
| GasLat_right_Dm | -0,166 | 0,250 |
| GasLat_right_Td | -0,093 | 0,522 |
| GasMed_right_Tc | 0,089 | 0,537 |
| GasMed_right_Ts | 0,082 | 0,570 |
| GasMed_right_Tr | 0,052 | 0,721 |
| GasMed_right_Dm | -0,027 | 0,854 |
| GasMed_right_Td | -0,102 | 0,484 |

| **Parametric correlations between left DKV and left TMG parameters** | | |
| --- | --- | --- |
|  | Pearson Correlation | Sig. |
| Gmax_left_Tc | 0,216 | 0,140 |
| Gmax_left_Ts | 0,104 | 0,483 |
| Gmax_left_Tr | -0,204 | 0,165 |
| Gmax_left_Dm | -0,009 | 0,952 |
| Gmax_left_Td | -0,254 | 0,081 |
| BF_left_Tc | -0,123 | 0,406 |
| BF_left_Ts | 0,271 | 0,063 |
| BF_left_Tr | -0,129 | 0,381 |
| BF_left_Dm | -0,252 | 0,084 |
| BF_left_Td | -0,258 | 0,076 |
| ST_left_Tc | 0,039 | 0,791 |
| ST_left_Ts | 0,183 | 0,214 |
| ST_left_Tr | -0,200 | 0,173 |
| ST_left_Dm | -0,125 | 0,397 |
| ST_left_Td | -0,236 | 0,106 |
| GasLat_left_Tc | 0,093 | 0,531 |
| GasLat_left_Ts | 0,096 | 0,517 |
| GasLat_left_Tr | -0,128 | 0,387 |
| GasLat_left_Dm | 0,034 | 0,817 |
| GasLat_left_Td | -0,268 | 0,066 |
| GasMed_left_Tc | -0,046 | 0,758 |
| GasMed_left_Ts | 0,126 | 0,394 |
| GasMed_left_Tr | -0,030 | 0,838 |
| GasMed_left_Dm | -0,016 | 0,914 |
| GasMed_left_Td | -0,179 | 0,223 |

| **Non-parametric correlations between left DKV and left TMG parameters** | | |
| --- | --- | --- |
|  | Spearman Correlation | Sig. |
| Gmax_left_Tc | 0,121 | 0,414 |
| Gmax_left_Ts | ,297^*^ | 0,041 |
| Gmax_left_Tr | -0,139 | 0,347 |
| Gmax_left_Dm | -0,062 | 0,674 |
| Gmax_left_Td | -0,195 | 0,184 |
| BF_left_Tc | -0,058 | 0,697 |
| BF_left_Ts | ,315^*^ | 0,029 |
| BF_left_Tr | -0,203 | 0,167 |
| BF_left_Dm | -0,233 | 0,112 |
| BF_left_Td | -,310^*^ | 0,032 |
| ST_left_Tc | 0,218 | 0,137 |
| ST_left_Ts | 0,260 | 0,075 |
| ST_left_Trm | -0,275 | 0,059 |
| ST_left_Dm | -0,037 | 0,804 |
| ST_left_Td | -,305^*^ | 0,035 |
| GasLat_left_Tc | 0,130 | 0,378 |
| GasLat_left_Ts | 0,275 | 0,059 |
| GasLat_left_Tr | -0,147 | 0,320 |
| GasLat_left_Dm | 0,081 | 0,586 |
| GasLat_left_Td | -0,247 | 0,091 |
| GasMed_left_Tc | 0,207 | 0,158 |
| GasMed_left_Ts | 0,180 | 0,222 |
| GasMed_left_Tr | 0,007 | 0,963 |
| GasMed_left_Dm | -0,057 | 0,701 |
| GasMed_left_Td | -0,180 | 0,222 |

| **Parametric correlations between right DKV and right MTT parameters** | | |
| --- | --- | --- |
|  | Pearson Correlation | Sig. |
| GMax_right_Tone | 0,181 | 0,212 |
| GMax_right_Stiffness | -0,029 | 0,842 |
| GMax_right_Elasticity | -0,085 | 0,564 |
| GMax_right_Relaxation | -0,148 | 0,310 |
| GMax_right_Creep | -0,109 | 0,454 |
| BF_right_Tone | -0,081 | 0,581 |
| BF_right_Stiffness | -0,102 | 0,487 |
| BF_right_Elasticity | -0,012 | 0,934 |
| BF_right_Relaxation | 0,136 | 0,352 |
| BF_right_Creep | 0,132 | 0,366 |
| ST_right_Tone | -0,096 | 0,510 |
| ST_right_Stiffness | -0,127 | 0,385 |
| ST_right_Elasticity | -0,086 | 0,555 |
| ST_right_Relaxation | 0,109 | 0,455 |
| ST_right_Creep | 0,103 | 0,482 |
| GasLat_right_Tone | -0,075 | 0,606 |
| GasLat_right_Stiffness | 0,007 | 0,964 |
| GasLat_right_Elasticity | 0,063 | 0,667 |
| GasLat_right_Relaxation | 0,024 | 0,872 |
| GasLat_right_Creep | 0,021 | 0,886 |
| GasMed_right_Tone | -0,103 | 0,483 |
| GasMed_right_Stiffness | 0,022 | 0,881 |
| GasMed_right_Elasticity | -0,020 | 0,891 |
| GasMed_right_Relaxation | -0,048 | 0,743 |
| GasMed_right_Creep | -0,068 | 0,643 |

| **Non-parametric correlations between right DKV and right MTT parameters** | | |
| --- | --- | --- |
|  | Spearman Correlation | Sig. |
| GMax_right_Tone | 0,206 | 0,156 |
| GMax_right_Stiffness | -0,051 | 0,728 |
| GMax_right_Elasticity | -0,089 | 0,545 |
| GMax_right_Relaxation | -0,153 | 0,293 |
| GMax_right_Creep | -0,196 | 0,178 |
| BF_right_Tone | -0,083 | 0,572 |
| BF_right_Stiffness | -0,101 | 0,488 |
| BF_right_Elasticity | -0,091 | 0,534 |
| BF_right_Relaxation | 0,117 | 0,425 |
| BF_right_Creep | 0,099 | 0,500 |
| ST_right_Tone | -0,130 | 0,374 |
| ST_right_Stiffness | -0,135 | 0,357 |
| ST_right_Elasticity | -0,161 | 0,269 |
| ST_right_Relaxation | 0,154 | 0,291 |
| ST_right_Creep | 0,142 | 0,330 |
| GasLat_right_Tone | -0,104 | 0,477 |
| GasLat_right_Stiffness | 0,031 | 0,832 |
| GasLat_right_Elasticity | 0,026 | 0,859 |
| GasLat_right_Relaxation | 0,064 | 0,660 |
| GasLat_right_Creep | 0,041 | 0,780 |
| GasMed_right_Tone | -0,111 | 0,449 |
| GasMed_right_Stiffness | 0,021 | 0,885 |
| GasMed_right_Elasticity | -0,042 | 0,775 |
| GasMed_right_Relaxation | -0,032 | 0,829 |
| GasMed_right_Creep | -0,059 | 0,689 |

| **Parametric correlations between left DKV and left MTT parameters** | | |
| --- | --- | --- |
|  | Pearson Correlation | Sig. |
| Gmax_left_Tone | 0.031 | 0.836 |
| Gmax_left_Stiffness | -0,093 | 0,532 |
| Gmax_left_Elasticity | 0,054 | 0,719 |
| Gmax_left_Relaxation | -0,211 | 0,154 |
| Gmax_left_Creep | -0,206 | 0,165 |
| BF_left_Tone | -0,190 | 0,201 |
| BF_left_Stiffness | -0,178 | 0,232 |
| BF_left_Elasticity | -,294^*^ | 0,045 |
| BF_left_Relaxation | 0,159 | 0,285 |
| BF_left_Creep | 0,154 | 0,302 |
| ST_left_Tone | -0,165 | 0,268 |
| ST_left_Stiffness | -0,180 | 0,225 |
| ST_left_Elasticity | -0,250 | 0,090 |
| ST_left_Relaxation | 0,159 | 0,286 |
| ST_left_Creep | 0,130 | 0,384 |
| GasLat_left_Tone | -0,097 | 0,518 |
| GasLat_left_Stiffness | -0,014 | 0,924 |
| GasLat_left_Elasticity | -0,046 | 0,759 |
| GasLat_left_Relaxation | 0,010 | 0,947 |
| GasLat_left_Creep | 0,016 | 0,915 |
| GasMed_left_Tone | -0,021 | 0,887 |
| GasMed_left_Stiffness | -0,001 | 0,993 |
| GasMed_left_Elasticity | -0,087 | 0,559 |
| GasMed_left_Relaxation | 0,008 | 0,956 |
| GasMed_left_Creep | 0,010 | 0,946 |

| **Non-parametric correlations between left DKV and left MTT parameters** | | |
| --- | --- | --- |
|  | Spearman Correlation | Sig. |
| Gmax_left_Tone | 0.029 | 0.775 |
| Gmax_left_Stiffness | -0,118 | 0,431 |
| Gmax_left_Elasticity | 0,031 | 0,836 |
| Gmax_left_Relaxation | -0,106 | 0,478 |
| Gmax_left_Creep | -0,092 | 0,539 |
| BF_left_Tone | -0,201 | 0,176 |
| BF_left_Stiffness | -0,173 | 0,244 |
| BF_left_Elasticity | -0,168 | 0,259 |
| BF_left_Relaxation | 0,134 | 0,370 |
| BF_left_Creep | 0,138 | 0,353 |
| ST_left_Tone | -0,136 | 0,363 |
| ST_left_Stiffness | -0,169 | 0,257 |
| ST_left_Elasticity | -0,177 | 0,233 |
| ST_left_Relaxation | 0,136 | 0,363 |
| ST_left_Creep | 0,108 | 0,470 |
| GasLat_left_Tone | -0,127 | 0,393 |
| GasLat_left_Stiffness | -0,084 | 0,573 |
| GasLat_left_Elasticity | 0,028 | 0,853 |
| GasLat_left_Relaxation | 0,055 | 0,713 |
| GasLat_left_Creep | 0,059 | 0,692 |
| GasMed_left_Tone | -0,044 | 0,768 |
| GasMed_left_Stiffness | -0,046 | 0,757 |
| GasMed_left_Elasticity | -0,085 | 0,572 |
| GasMed_left_Relaxation | 0,031 | 0,836 |
| GasMed_left_Creep | 0,024 | 0,873 |
